# Can evolutionary theories of dispersal and senescence predict postrelease survival, dispersal, and body condition of a reintroduced threatened mammal?

**DOI:** 10.1002/ece3.7115

**Published:** 2020-12-30

**Authors:** Natasha M. Robinson, Wade Blanchard, Christopher MacGregor, Rob Brewster, Nick Dexter, David B. Lindenmayer

**Affiliations:** ^1^ Fenner School of Environment and Society The Australian National University Canberra ACT Australia; ^2^ The National Environmental Science Program Threatened Species Recovery Hub Fenner School of Environment and Society, The Australian National University Canberra ACT Australia; ^3^ Rewilding Australia Rose Bay NSW Australia; ^4^ Booderee National Park Jervis Bay JBT Australia

**Keywords:** aging, body mass or weight, conservation translocation, Dasyuridae, dispersal theory, endangered or threatened species, reintroduction, survival, theory of senescence

## Abstract

1. Theories of dispersal and senescence (or aging) predict that dispersal, and ongoing survival and body condition, are influenced by evolutionary drivers, along with intrinsic and extrinsic factors. Such theories are relevant to translocations of animals where high mortality, loss of body condition, and dispersal beyond the area of release are commonly reported. However, these theories have rarely been tested using data from translocations.
2. We explore whether theories of dispersal and senescence, together with biological knowledge and management interventions, can predict rates of postrelease dispersal, survival and change in body condition of a translocated endangered meso‐predator, the eastern quoll *Dasyurus viverrinus*.
3. Captive‐bred quolls (*n* = 60) from three sanctuaries were translocated to an unfenced, predator‐managed reserve (Booderee National Park) over 2 years (2018, 2019). Survival, dispersal and body mass were monitored via GPS/VHF tracking and targeted trapping for 45 days postrelease.
4. We found support for the “social subordinate” hypothesis, with smaller quolls dispersing further. Consistent with theories of senescence and the biology of our species, survival was marginally greater for females, and females regained losses in body mass in both years following release. In contrast, males recovered body condition in the first but not the second release as this coincided with breeding. Quolls that originated from the mainland sanctuary were on average heavier at release and, after accounting for weight, dispersed further.
5. *Synthesis and applications*. Using theory to test outcomes of wildlife translocations can provide insights into patterns across taxa and under different conditions, enabling useful improvements to future fauna translocations. This allows for better predictions to be made about the likelihood of success from proposed translocations, changes to planning to improve outcomes (e.g., modifying sex ratios, individual selection and release cohort), and improved animal welfare as fewer animals are subjected to trials.

## Graphical abstract

We test the applicability of theories of dispersal and senescence (or ageing) in predicting postrelease dispersal, survival, and change in body condition of translocated fauna; we use the eastern quoll as a case study. We found support for the “social subordinate” hypothesis, with smaller quolls dispersing further. Consistent with theories of senescence and our species biology, survival was marginally greater for females, and females regained losses in body mass in both years following release. Integrating theory into practice enables improved understanding of species responses to management actions such as fauna translocations.

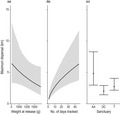

## 1 INTRODUCTION

Animal translocations are implemented for different purposes (e.g., conservation, economic, social goals, IUCN/SSC, 2013). *Reintroductions* aim to re‐establish species in areas they once occupied, *introductions* establish species in new areas, and *supplementations* or are used to bolster existing populations. Animal translocations are often plagued by low survival rates and, where there are no barriers (e.g., islands, fenced reserves), high levels of dispersal beyond the area of release (Le Gouar et al., 2012). Translocations of captive‐bred animals to the wild often have higher rates of mortality compared to wild‐to‐wild translocations as captive‐bred animals have less experience with foraging, finding shelter and are more naïve to threats in the wild (Fischer & Lindenmayer, 2000; Jule et al., 2008). Yet, captive‐bred individuals are often used for conservation‐orientated translocations, especially for endangered animals, as wild source populations are small or nonexistent (Jule et al., 2008). It is important to consider how to improve the success of animal translocations as such operations are resource intensive and ethically questionable if not justified by good planning, monitoring, threat mitigation, and evaluation (e.g., IUCN/SSC, 2013; Robinson et al., 2020). Central to improving animal translocations is understanding what factors contribute to enhanced survival and establishment in the released area.

Minimal dispersal, ongoing survival, and evidence of breeding are characteristics of successful early establishment of a translocated population (e.g., Moseby et al., 2011; Robinson et al., 2018). Dispersal in its broadest sense refers to unidirectional movement from one habitat patch to another (Bowler & Benton, 2005). Dispersal can be further broken down into three sequential behavioral stages (departure, transience, settlement, Clobert et al., 2009). Following release into a new environment, translocated animals are often found to disperse away from a release point (Le Gouar et al., 2012; Stamps & Swaisgood, 2007). Large movements can compromise the success of a program if animals fail to establish at the release location (Le Gouar et al., 2012; Robinson et al., 2020). At the individual level, overdispersal can hinder breeding as chances of interacting with conspecifics declines (Le Gouar et al., 2012). The higher energy demands and stress of constant movement or long‐distance dispersal can further compromise body condition and survival (Moehrenschlager & Macdonald, 2003). Animals in poor body condition or enduring stress, in turn, have reduced breeding success (Atkinson & Ramsay, 1995; Facka et al., 2016). Managers typically try to minimize dispersal by using different tactics (e.g., release method, supplementary feeding, Batson et al., 2015; Le Gouar et al., 2012). Conversely, site fidelity can be detrimental to translocated animals if, for example, the habitat is unviable or inadequate, or the concentration of animals leads to predators being attracted to a release site (e.g., via novel scents, Banks et al., 2002). Consequently, dispersal may be a natural response by an individual to survive (Bennett et al., 2013). Dispersal, survival, and changes in body condition following release are regularly investigated for animal translocations (e.g., Bright & Morris, 1994; Le Gouar et al., 2012). Various authors have designed their studies according to intrinsic factors such as sex, age and personality (e.g., Germano et al., 2017). Other studies provide post hoc explanations for differences in outcomes as a function of these intrinsic factors, typically interpreting findings as consistent with life history strategies or social organization (e.g., Letty et al., 2000). To our knowledge, no one has attempted to develop a broad and predictive theoretical framework to guide management decisions for translocations. Our goal here is to move forward the field of translocation biology by articulating how evolutionary theories can be integrated into translocation planning and predictions.

Incorporating theory into translocation practice can improve outcomes for translocations. Many hypotheses have been generated from theory to describe patterns in dispersal, body condition, and survival (or conversely, mortality, and senescence) (e.g., Greenwood, 1980, Kirkwood, 1977, Williams, 1957, and reviews by Bowler & Benton, 2005, Lawson Handley & Perrin, 2007, Lemaître et al., 2015 with summaries therein). However, rarely have these theories and hypotheses been tested or applied in the context of translocations (but see Stamps & Swaisgood, 2007). According to theory, dispersal is often sex‐biased as determined by the type of mating system (Greenwood, 1980), or resource competition (Trochet et al., 2016, table 1). Greenwood (1980) proposed dispersal would be male‐biased when, during breeding, the male does not retain exclusive access to one or more females, there is male‐male aggression, and where females invest disproportionately in reproduction. Trochet et al. (2016) describe other factors that could cause sex‐biased dispersal that are linked to, but not necessarily caused by, mating systems per se (e.g., parental care, sexual dimorphism). Shaw and Kokko (2014) further proposed that sex‐biased dispersal was associated with timing relative to breeding, with greater dispersal expected during breeding.

The “social subordinate hypothesis” explains mechanisms relating to individual fitness and behavior and further predicts that body condition and relative size of individuals influences dispersal (Christian, 1970, table 1). Sexual dimorphism in size further promotes dispersal of the larger sex (Trochet et al., 2016). According to theories of senescence, sex‐biased survival and differences in body condition are expected when one sex engages in energetically costly behavior such as fighting (Clutton‐Brock & Isvaran, 2007; Lemaître et al., 2015). Not all theories or associated hypotheses of dispersal and senescence are transferable to animal translocations as different factors may drive responses. For examples, dispersal theories that relate to habitat saturation are less relevant until the population reaches carrying capacity (Bowler & Benton, 2005). Theories of dispersal and senescence, however, can help understand evolutionary causes driving different responses in translocated animals (Bowler & Benton, 2005). Translocations may further be used to experimentally test theories and/or hypotheses that are not easily examined under natural conditions (Gundersen et al., 2002).

In this paper, we test the applicability of theories of dispersal and senescence to wildlife translocations. We use the translocation of an endangered meso‐predator, the eastern quoll *Dasyurus viverrinus,* as a case study. We specifically asked: Is postrelease dispersal related to sex, timing of release relative to breeding, sex‐adjusted body mass, and or source of individuals? Is survival related to sex, timing, generations‐in‐captivity, and or source? Is body condition related to sex, timing, and or source? We used theory, along with knowledge of our study species' biology and other translocation studies, to make several predictions. We predicted that dispersal would be: (1) sex‐biased and greater for males; (2) higher in the second year (i.e., during the breeding season); and (3) greater for smaller individuals (Table 1). We predicted that survival would be: (4) sex‐biased and higher for females; (5) greater in the second year, following improved management of threats; and (6) higher for animals with fewer generations‐in‐captivity. Regarding body condition, we predicted that (7) females would regain mass more quickly, and that (8) recovery of body condition would be less in the second year due to timing of release during breeding (Table 1).

**TABLE 1:** Predictions for dispersal, survival, and body condition for translocated captive‐bred eastern quolls based on theory, biology, and management

	Theory and or mechanism	Eastern quoll biology and management tactics	Predictions for translocated quolls
Dispersal	Dispersal is sex‐biased as determined by the type of mating system, or resource competition. In polygamous mammals, and where there is strong competition among males for mates and local resources are important for females, dispersal is primarily male‐biased (Greenwood, 1980; Trochet et al., 2016)	Quolls are promiscuous breeders, with predominantly male‐biased natal dispersal (Godsell, 1983). During the breeding season, aggression between males increase (Godsell, 1983)	Males will disperse further than females
	Sex‐biased dispersal is influenced by timing relative to breeding. Male dispersal is predicted to be greater when dispersal concurrent with breeding (Shaw & Kokko, 2014)	During the breeding season, male quolls move more (Godsell, 1983). In 2018, quolls were released prior to breeding. In 2019, quolls were released six weeks later, coinciding with the breeding season	Dispersal of males will be greater in 2019 than in 2018
	Dispersal is related to body condition and size. Negative interactions between conspecifics causes smaller, less aggressive, and/or younger subordinates to disperse (Christian, 1970)	All quolls were released with minimum body mass that is greater than juvenile dispersal mass Quolls are sexually dimorphic with males heavier than females (Godsell, 1983)	Accounting for sex, smaller quolls will disperse further than larger quolls
Survival	Survival and longevity is sex‐biased in polygamous, sexually dimorphic mammals. Promiscuous mating and sexual dimorphism favoring larger males is associated with increased male competition for breeding. Energetically costly traits reduce longevity, resulting in differences in survival rates between sexes (Clutton‐Brock & Isvaran, 2007; Lemaître et al., 2015; Williams, 1957)	Male quolls have reduced survival during and following the breeding season (Godsell, 1983); this is related to behavioral and physiological changes (e.g., increased testosterone levels)	Females will have a higher survival rate than males following release
	Targeted management interventions combined with monitoring and evaluation enables improved conservation outcomes (Robinson, Scheele, et al., 2018; Scheele et al., 2018)	Following knowledge gained about threats to quoll survival in 2018, changes were made to subsequent translocation procedures and management in 2019 (Robinson et al., 2020)	Survival will be higher in 2019 than in 2018
	Generations‐in‐captivity is related to loss of native instincts and behaviors required for survival in the wild; longer generations‐in‐captivity leads to reduced survivorship (McPhee, 2004)	In a related species, the Tasmanian devil *Sarcophilus harrisii*, increasing generations‐in‐captivity was linked to increased mortality upon release into the wild (Grueber et al., 2017)	Survival will be greater for animals with lower generations‐in‐captivity
Body condition	Translocation causes loss of body condition as animals adjust to new environment (Bright & Morris, 1994; Teixeira et al., 2007). Energetically costly traits result in differential rates of recovery of body condition between sexes; individuals optimize resource allocation between body growth, repair and reproduction according to early‐late trade‐offs in reproduction and longevity (Clutton‐Brock & Isvaran, 2007; Kirkwood, 1977; Lemaître et al., 2015)	During the breeding period, both male and female quolls lose weight but males lose more proportionally (Godsell, 1983)	Animals will lose body weight following translocation and then regain it over time; recovery will be faster for females than males
	Timing of release influences change in body condition postrelease (Bright & Morris, 1994)	Body mass of both sexes increase prior to breeding, then decline during breeding (Godsell, 1983). The timing of the 2019 release during breeding will mean that females will have less opportunity to regain body mass	Recovery of body mass will be less in 2019 than 2018; breeding success will be lower in 2019 than 2018

## 2 MATERIALS AND METHODS

### 2.1 Study area

**FIGURE 1:**
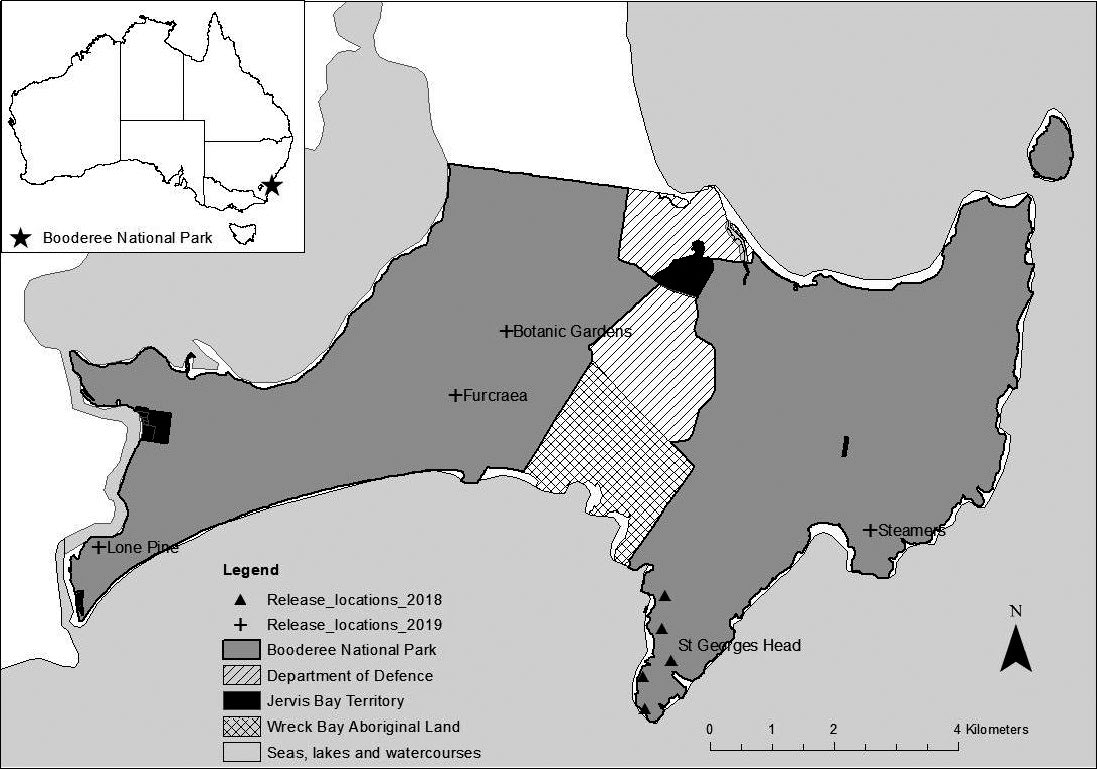
Booderee National Park (BNP) with adjacent land tenure; inset map shows the location of BNP as indicated by the star. Eastern quolls were released in the park at five locations in 2018 and 2019

Our study was conducted at Booderee National Park (BNP), a 6,400 ha unfenced reserve on the south‐east coast of Australia (35°10′S, 150°40′E, Figure 1). The park is co‐managed by Parks Australia and the Traditional Owners of the land, the Wreck Bay people (DNP, 2015). The park receives an average annual rainfall of ~1,243 mm (BOM, 2019b), with a mean monthly temperature range of 15–26°C (BOM, 2019a). Vegetation ranges from open forest, rainforest, and woodland through to heath, shrub, sedge, and swamps. The park conducts invasive species control (primarily for the feral predator, the red fox *Vulpes vulpes*, and invasive bitou bush *Chrysanthemoides monilifera* ssp. *monilifera*).

### 2.2 Study species

The eastern quoll *Dasyurus viverrinus* (hereafter quoll) is a small‐medium sized marsupial predator. The species is sexually dimorphic (females 700–1,100 g; males 900–2,000 g) and occurs in two distinct color morphs occur, black, or fawn. Quolls are promiscuous breeders (Godsell, 1983) and in related species, *Dasyurus maculatus*, multiple parentage is possible in a single litter (Glen et al., 2008). Breeding commences in May‐June each year, with births occurring in June‐July (Godsell, 1983). Juveniles are reliant on maternal care until five to six months old.

The eastern quoll was once common throughout south‐eastern Australian states, including Tasmania (Peacock & Abbott, 2014; Robinson et al., 2020). Prior to reintroduction to BNP, the last confirmed sighting of a wild quoll alive on mainland Australia was in 1963 (Dickman et al., 2001). The species still persists in Tasmania, although populations are declining (Fancourt, 2016). Declines and extinctions on mainland Australia are attributed to a combination of disease and predation by the introduced red fox (Peacock & Abbott, 2014). Predation by the red fox, as well as the domestic dog *Canis familiarus*, continue to threaten the re‐establishment of the quoll on the mainland, along with collisions with vehicles (Robinson et al., 2020). The species is listed nationally and internationally as Endangered (Australian Government 2020, Burbidge & Woinarski, 2016).

### 2.3 Translocation

We reintroduced 60 eastern quolls to BNP over 2 years, beginning with 20 quolls in March 2018, and a further 40 quolls in April–May 2019 (see Table S1 for further details of release). We sourced quolls from two captive breeding facilities in Tasmania (Devils@Cradle, Trowunna Wildlife Sanctuary) and a third sanctuary located on mainland Australia (Aussie Ark). All three sanctuaries share breeding animals and aim to maximize genetic diversity of stock and minimize generations in captivity. We selected animals according to minimum criteria of health, body mass, and breeding potential (for details see Robinson et al., 2020).

The timing of the release took into account predicted threats to re‐establishing quolls in the park, notably minimizing the risk of mortality from paralysis tick *Ixodes holocyclus* envenomation (Robinson et al., 2020). In 2019, the release was delayed a further six weeks to minimize quoll fatalities from vehicular collisions during periods of high visitor traffic. In 2018, we released all quolls in the same broad location (five sites, ~500 m apart; Figure 1). In the second year, we changed management tactics and released animals in sanctuary groups at four locations over two days (Figure 1); this was to maximize existing social cohesion within sanctuary groups and minimize competition for resources (e.g., Shier & Swaisgood, 2012).

### 2.4 Monitoring

Prior to the release of quolls, we fit GPS/VHF collars to all 20 animals released in 2018 (Telemetry Solutions, model FLR V LS14250) and 21 of the 40 animals released in 2019 (Sirtrack, model ZV6C 163 Zilco VHF Collar). Both types of collars contained a mortality signal. In 2019, we selected animals for collaring that reflected an even sex ratio, representation of each sanctuary, and a range of behavioral traits (e.g., bold/shy) observed during captivity. We tracked all collared quolls daily for the first 4 weeks after release, then 3–4 times per week until day 45. We were restricted to this period of monitoring by the relocation of quolls in 2018 at day 45 that interrupted their movement patterns (Robinson et al., 2020). We conducted targeted trapping every 2–3 weeks throughout the 45 day period to check on body mass, collar fit, signs of pouch young, and general health. We also used camera traps at supplementary feed stations and incidental sightings to record locations of all animals, not only those with collars. Our regular tracking and intensive monitoring ensured rapid detection of mortalities and, combined with necropsies, allowed a cause of death to be determined. Research was approved by The Australian National University Animal Experimentation Ethics Committee (protocols A2016/30, A2018/71).

### 2.5 Statistical analyses

We analyzed dispersal, survival, and body condition over a 45‐day period postrelease for 2018 and 2019 according to sex, year, generations‐in‐captivity (survival only), sanctuary of origin (Devils@Cradle, Trowunna, Aussie Ark), and sex‐adjusted release weight (survival, dispersal only). We included sex‐adjusted weight as sexual dimorphism in quolls is expected to promote sex‐biased dispersal and survival (Trochet et al., 2016). Sex‐adjusted release weights were computed using *z*‐scores, that is, where we assumed a common standard deviation for both sexes and a different mean for each sex. Generations‐in‐captivity was calculated as the average generations‐in‐captivity of the parents plus one (Grueber et al., 2017; ISIS, 2005). We report 95% credible intervals for all models and refer to effects as being significant when their 95% credible intervals do not include zero (the null value). We refer to effects as marginal when their 90% credible intervals do not include zero but overlapped zero at 95%; in this instance, we report both the 90% and 95% credible intervals. See Supporting Information S1 for terms used in analyses and Tables S2–S4 for the full list of models.

We calculated individual maximum dispersal as the straight‐line displacement between its point of release and most distant location where it was recorded (White & Garrott, 1980). We analyzed maximum dispersal accounting for number of tracking days (or survival days), as the longer an animal was tracked (or survived), the further it was able to move. We included data only for daytime tracking locations of animals that were released with a collar, as this was consistent across years. If a quoll was found predated, we excluded this last location. We modeled maximum dispersal distance using a Bayesian Generalized linear model with a Gamma distribution and log link function and fit it using the brms R package (Bürkner, 2017, 2018). We used default priors, which consist of a flat (improper) prior for the regression parameters, a student‐*t* prior for the intercept with 3 degrees of freedom with location of 1 and scale of 10 and a gamma prior (0.01, 0.01) for the shape parameter. We ran four Markov chains for 2,000 iterations with a burn‐in of 1,000 yielding 4,000 samples for posterior inference. We assessed the convergence of all parameters with the Gelman‐Rubin diagnostic (Gelman & Rubin, 1992), denoted by $\hat{R}$, all parameters meet the convergence criteria of $\hat{R}<1.01$. We performed model selection over the 16 possible models main effect models and one additional model that included the interaction between sex and year and all main effects (see Table S2 for a complete list of models). We chose the model with the lowest widely applicable information criteria (WAIC) (Vehtari et al., 2017) for further inference.

We calculated survival status of all quolls up to 45 days following release. If an individual was not seen following release, we excluded it from the analysis. All individuals that survived more than 45 days were right‐censored. We analyzed survival using Bayesian Cox proportional hazards model (Collett, 2003) in the INLA package (Martins et al., 2013) in R version 3.5.1 (R Core Team, 2018). We used the default priors in INLA for all model parameters, which are normal priors with mean zero and variance 1,000. We chose the best fitting model according to the WAIC from among the 33 models (see Tables S3). Estimated survival curves were computed via the Kaplan–Meier method (Collett, 2003).

We conducted growth curve analysis on body weights over time. We used the release weight at BNP as the baseline measure and subsequent weights of animals caught in traps during the 45 day period. We implemented a Bayesian multi‐level model using the brms package (Bürkner, 2017, 2018) with a random effect for individual. We assumed that weights followed a Gaussian distribution and the default priors which are similar to the maximum dispersal model with the exception that the variance components have half *t*‐distributions with 3 degrees of freedom and scale of 271. We used WAIC to choose the best fitting model from the list supplied in Table S4a.

## 3 RESULTS

Translocated male quolls were heavier than females upon release, with males weighing on average 1,294 g and females 854 g. Generations‐in‐captivity ranged from 1 to 3.99 (average 3.19). Within 45 days of release, the dispersal range of male quolls was between 247 and 9,481 m, and for female quolls 263 and 6,986 m. During this same period, 40% of quolls in 2018 (8 of 20) and 45% of quolls in 2019 (15 of 33) were known to have survived. The fate of seven quolls in 2019 remained unknown. The main causes of death were predation by fox and dog (*n* = 19) and collision with motor vehicles (*n* = 7). In the first year, all three surviving females bred, with evidence of pouch young. However, in the second year, only half of the 12 surviving females had pouch young.

### 3.1 What factors are important for dispersal?

**FIGURE 2:**
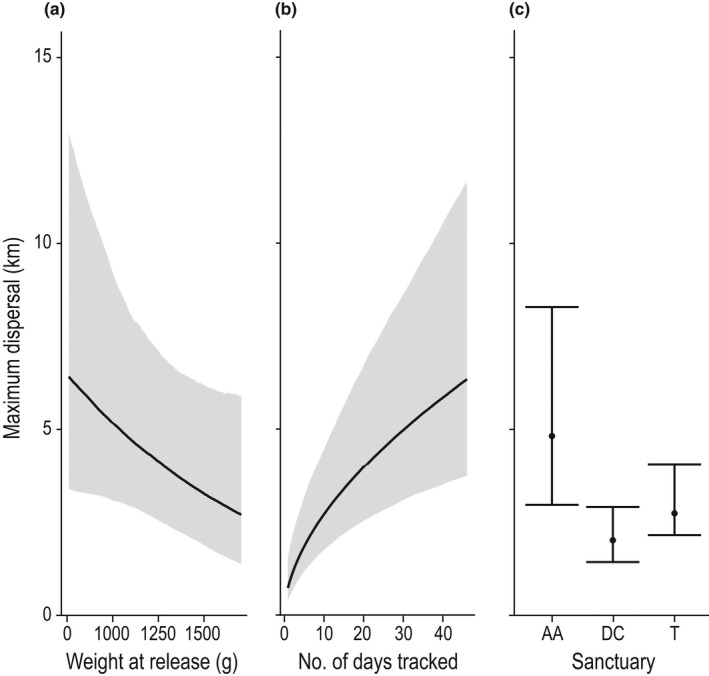
Maximum dispersal of translocated eastern quolls at 45 days since release according to (a) release weight; (b) number of days tracked; and (c) sanctuary of origin. 95% credible intervals are shown by the gray shading (a, b) and bars (c)

We found support for only one of our predictions based on dispersal theory; accounting for sex, smaller quolls dispersed further than larger quolls (Table 1: prediction 3, Table 2, Figure 2a, Table S2). Unsurprisingly, dispersal also increased the longer an animal was tracked (or remained alive), and was greater for quolls originating from Aussie Ark sanctuary than the other two sanctuaries (Table 2, Figure 2b,c, Table S2). There was no significant difference in dispersal distances between sexes or years (Table 1: predictions 1, 2).

**TABLE 2:** Model coefficients for best fitting model for maximum dispersal, with 95% credible intervals (CI). Sanctuary (Aussie Ark) is the baseline for sanctuary dispersal differences

Coefficient	Estimate	Lower 95% CI	Upper 95% CI
Intercept	−0.19	−0.81	0.55
Weight	−0.30	−0.57	−0.03
Log Number of Days Tracked	0.56	0.34	0.77
Sanctuary (Devils@Cradle)	−0.98	−1.69	−0.31
Sanctuary (Trowunna)	−0.78	−1.47	−0.12

### 3.2 What factors are important for survival?

**FIGURE 3:**
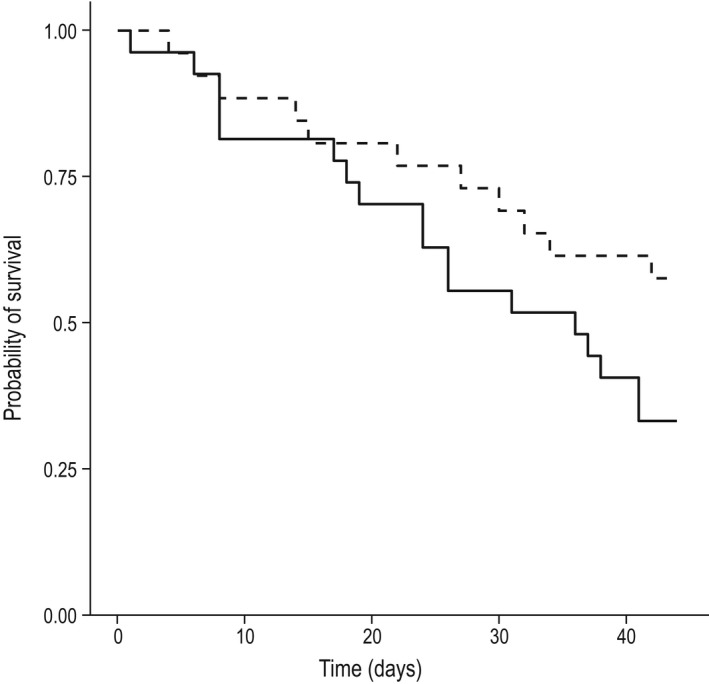
Kaplan–Meier survival curves for male and female quolls over a 45 day period since release

We found partial support for our fourth prediction (Table 1). Consistent with senescence theory, females had a marginally higher rate of survival than males (estimate 0.61), with 95% credible intervals including zero (0.12–1.38) but not for 90% intervals (0.015, 1.25; Figure 3). Model selection results indicated that only a marginal effect of sex was needed in the model (Table S3). No difference in survival was found between years, or generations‐in‐captivity (Table 1: predictions 5, 6), or other predictor variables (body mass, sanctuary of origin).

### 3.3 What factors are important for recovery of body condition?

**FIGURE 4:**
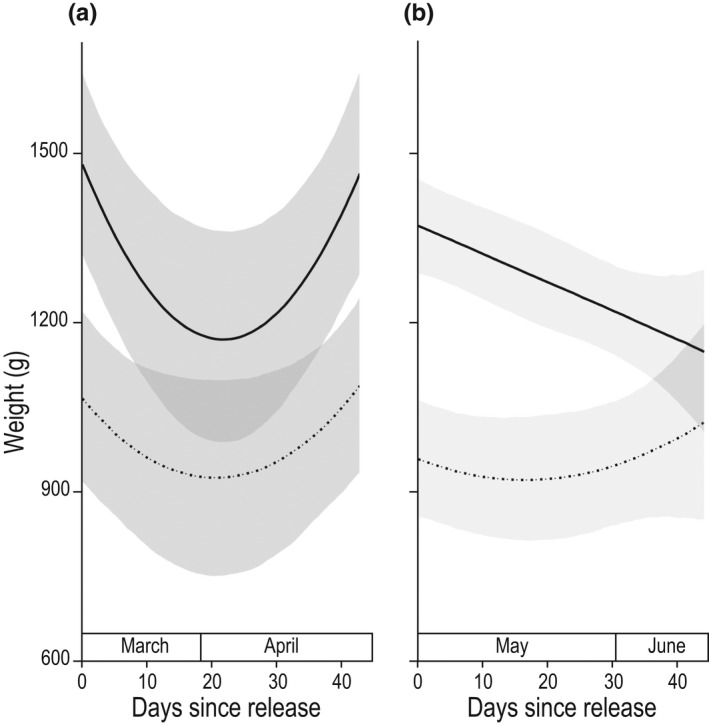
Postrelease weight change of translocated eastern quolls over a 45‐day period according to year of release (a: 2018, b: 2019) and sex (female: dotted line, male: solid line)

Postrelease body condition was influenced by sex, year (Figure 4, Table 1: predictions 7, 8) and sanctuary (Table S4a,b). Multi‐level model selection for body mass contained evidence of a 3‐way quadratic relationship between sex, year, and days‐since‐release on the change in weight by quolls (Figure 4, Table S4a,b). In both years, body weights declined for males and females following release (Figure 4). However, only females recovered lost body condition in both years (Table 1: prediction 7). Differences in timing of release affected recovery of body condition between years (Table 1: prediction 8). In 2018, both male and female weights declined postrelease and then recovered to similar release weights within the 45 day study period. In 2019, male weights declined with no recovery and female weights declined then surpassed their initial weight over the monitoring period. Quolls from Aussie Ark were heavier at release (on average 200 g more) than animals from the other two sanctuaries (Table S4b).

## 4 DISCUSSION

We investigated the applicability of theories of dispersal and senescence to faunal responses following translocation. We used theory, along with biological knowledge of our study species and expected response to management intervention, to predict differences in dispersal, survival and change in body condition following release. We found support for half of our predictions, with theory and biological knowledge predicting responses for dispersal based on body size; survival according to sex; and differences in recovery of body condition between sexes and years (Table 1: predictions 3, 4, 7, 8). Evolutionary theories of dispersal and senescence may be useful in predicting animal responses following translocation, but forecasts are likely to be species‐specific and subject to other intrinsic (e.g., individual behavior) and extrinsic factors (e.g., competition, predation). Biological knowledge of species remains an important component of responses by translocated fauna in novel environments.

### 4.1 Postrelease dispersal is determined by size, but not sex or season

Dispersal is expected to be male‐biased in mammals as a consequence of their mating system and resource competition (Greenwood, 1980; Trochet et al., 2016). Dasyurids, the marsupial family that includes our study species, are promiscuous breeders, engage in male‐male aggression during breeding, and exhibit maternal care; accordingly, quoll dispersal is predominately male‐biased (Godsell, 1983). However, we found no evidence to suggest that dispersal of quolls following translocation was male‐biased. We predicted sex‐biased dispersal to be even more pronounced in the second year, with the timing of release coinciding with breeding and heightened male competition (Shaw & Kokko, 2014). However, again, we found no difference in dispersal between sexes. It is possible that males restricted their movements to areas where females were located to ensure mating. Both sexes were released together, and thus, males were able to follow individual movements or scent trails (Godsell, 1983). It is also possible that tracking for longer periods might have revealed different patterns of dispersal. Our tracking was limited to 45 days; thus, quolls might have been still in the exploring or transience phase of dispersal. It might otherwise be possible that postrelease dispersal is not influenced by the same factors that drive natal dispersal (Lawson Handley & Perrin, 2007; Richardson & Ewen, 2016). Translocations are enforced displacement in unfamiliar territory; this can prompt dispersal away from the release point, especially if important cues are not present (e.g., habitat, Stamps & Swaisgood, 2007). In addition, social factors such as the presence of conspecifics (Richardson & Ewen, 2016) and or social dominance may motivate dispersal more than sex or season.

The social subordinate hypothesis predicts that smaller, less socially dominant individuals will be forced to disperse when faced with larger, more dominant individuals (Christian, 1970). Our data were consistent with this hypothesis, with smaller quolls (after we controlled for sex) dispersing further. In the wild, quolls are considered solitary predators; their territories overlap but they tend to avoid other individuals and thus have minimal social interaction (Godsell, 1983). Interactions that occur are mainly agonistic, with aggression between males increasing during breeding and between females when carrying large pouch young (Godsell, 1983). We found smaller individuals dispersed further, consistent with the social subordinate hypothesis, yet distances were still within known movement ranges (Jones, 2000). Thus, any negative interactions resulting in dispersal, did not result in long‐distance movements. At higher densities, or in more territorial species, or within groups of unfamiliar individuals, intra‐sexual competition based on size may result in longer dispersal distances (Shier & Swaisgood, 2012).

We found that dispersal varied between animals sourced from different sanctuaries, with animals from Aussie Ark dispersing further than the other two sanctuaries. Rogers et al. (2016) posited that environmental factors between captive breeding facilities could influence behaviors of released animals. Although all three sanctuaries involved in our study have a similar management style (e.g., minimal handling, natural features and vegetation), there are differences in climatic zones, with Aussie Ark the only mainland sanctuary. Another explanation for greater dispersal of quolls originating from Aussie Ark is that the two sites where these animals were released had less favorable habitat than the other sites (Le Gouar et al., 2012) or were missing important habitat cues (Stamps & Swaisgood, 2007). However, we cannot disentangle these effects as we did not have sanctuary replication at all sites. In the first year, we released Devils@Cradle and Trowunna animals at both release points in equal male:female pairs. In the second year, to maximize social cohesion based on their captive breeding experience (Shier & Swaisgood, 2012), we released animals in sanctuary groups at single sites. We further used multiple release locations to minimize potential overdispersal due to crowding and competition for resources such as dens (Godsell, 1983).

Dispersal could also have been affected by other intrinsic factors such as individual behavioral traits and extrinsic factors such as supplementary feeding and environmental cues (Berger‐Tal et al., 2019; Le Gouar et al., 2012; Stamps & Swaisgood, 2007). We provided supplementary feeding at release points to minimize dispersal and mitigate losses of body condition (Jule et al., 2008). Translocated quolls remained within normal dispersal range of their release location and frequented feeding stations indicating that this strategy worked; but it may have also diminished the strength of our predictions.

### 4.2 Females have greater levels of survival than males

Theory of senescence predicts that investment in energetically costly traits in one sex will result in differential survival rates (Kirkwood, 1977; Williams, 1957). We found support for this theory, with translocated female quolls having marginally greater survival than their male counterparts. This aligns with knowledge of the species' biology and behavior. Male quolls engage in fighting and undergo physiological changes during the breeding season which is associated with reduced survival (Godsell, 1983). In other smaller Dasyurids (e.g., *D. hallucatus, Antechinus* spp), mating is followed by partial to complete male die‐off (Dickman & Braithwaite, 1992). As the eastern quoll is a promiscuous breeder, translocations of sufficient numbers of males to compensate for male‐biased mortality will be necessary for ensuring adequate breeding opportunities and establishing populations.

We found no evidence that management tactics increased survival of translocated captive‐bred quolls; survival did not change with increasing generations‐in‐captivity or due to timing of release to reduce road fatality. Overall, we observed an increase in numbers of quolls surviving in 2019, and the rate of known vehicle collisions more than halved between years from 20% in 2018 to 7.5% in 2019, but survival rates only marginally improved. This could be due to the range in our data being too small to generate differences (e.g., generations‐in‐captivity), or limited ability for management tactics to influence other extrinsic factors (e.g., road fatality vs. predation).

Increasing generations‐in‐captivity was strongly associated with increased vulnerability to fatal collisions with vehicles in translocated captive‐bred Tasmanian devils, a related Dasyurid, although this effect was weaker when wild‐born individuals were removed from analysis (Grueber et al., 2017). Comparatively, the range in our data was smaller and did not include wild‐born quolls. In addition, road fatality was not the only threat to quoll survival, with predation (mainly by the red fox and domestic dog) causing more than twice as many fatalities as collisions with vehicles. Future translocations could source individuals from the wild, as they are likely to have greater survival skills than captive‐bred individuals (Jule et al., 2008). However, wild individuals are harder to source and their removal may compromise established wild populations, especially for threatened species (Todd et al., 2002). Wild quolls from Tasmania are not expected to have an appropriate predator response to foxes on the mainland (Jones et al., 2004); this is due to foxes being absent or occurring at very low densities in Tasmania (Ramsey et al., 2018) and a lack of co‐evolution of the two species (Woinarski et al., 2015).

### 4.3 Recovery of body condition postrelease varies between sexes and with timing of release

Translocations often result in animals losing condition postrelease (Bright & Morris, 1994), with captive‐bred mammalian carnivores being at particular risk of starvation (Jule et al., 2008). Our data support this with both sexes initially losing body mass in both years. However, differential rates of recovery between sexes are expected when one sex engages in more energetically costly activities for the benefit of reproducing (Clutton‐Brock & Isvaran, 2007; Kirkwood, 1977; Williams, 1957). As predicted, we found differences in recovery of body mass between sexes and between years due to timing of release concurrent with breeding.

Theory of senescence suggests that there are early‐late life trade‐offs that can result in differential rates of recovery of body condition to maximize reproduction (Kirkwood, 1977; Williams, 1957). In the eastern quoll, these trade‐offs are reflected in differences in physiology and behavior between sexes (e.g., during breeding, male quolls decline in body condition while females normally increase, Godsell, 1983). Elevated male‐male combat, combined with the energetic costs of breeding and stress associated with translocation (Teixeira et al., 2007), likely prevented recovery of male body condition within the period of monitoring. In contrast, females were able to recover body condition across both years. However, in the second year, as breeding occurred soon after release, recovery of body condition and or breeding success may have been compromised (Atkinson & Ramsay, 1995). Similarly, Facka et al. (2016) found that the late release of translocated female fishers (*Pekania pennanti*) resulted in a lower denning rate than those released early. We recommend that future translocations take account of seasonal physiological changes and periods of increased activity when considering the timing of release.

## 5 CONCLUSION

We used theories of dispersal and senescence along with biological knowledge to make several predictions regarding dispersal, survival, and body condition of translocated captive‐bred eastern quolls. We found support for half of our predictions. These were that dispersal decreases with body size; survival is greater for females; and females recover body condition more than males during the period observed. Evolutionary theory should be used in combination with species‐specific knowledge to understand and predict responses of translocated animals, and thereby improve translocation outcomes. Conservation managers can use these predictions to assess the likelihood of success of the translocation and or modify the program. For example, theory predicts that strong competition for mates and or resources can result in sex‐biased survival and dispersal. Managers can mitigate sex‐biased survival by adjusting the sex ratio of the release cohort, and sex‐biased dispersal might be lessened by releasing animals that are socially familiar together, minimizing conflict due to unfamiliarity (e.g., Shier & Swaisgood, 2012). Incorporating theory into practice can have animal welfare benefits as fewer animals are subjected to trials, and program modifications can enhance survival and establishment of translocated animals. Translocations are an increasingly important management tool for restoring populations (Berger‐Tal et al., 2019; Fischer & Lindenmayer, 2000). Translocations can be used to experimentally test and develop new theory and thus augment value of such management interventions beyond species restoration. This will enable broader trends in species responses to be predicted and lead to improved translocation design and procedures and ultimately better conservation outcomes.

## CONFLICT OF INTEREST

The authors declare no conflict of interest.

## AUTHOR CONTRIBUTIONS

**Natasha M. Robinson:** Conceptualization (equal); Data curation (lead); Formal analysis (supporting); Investigation (lead); Methodology (lead); Writing‐original draft (lead). **Wade Blanchard:** Formal analysis (lead); Methodology (supporting); Writing‐review & editing (equal). **Christopher MacGregor:** Data curation (supporting); Investigation (supporting); Writing‐review & editing (equal). **Rob Brewster:** Investigation (supporting); Writing‐review & editing (equal). **Nick Dexter:** Investigation (supporting); Writing‐review & editing (equal). **David B. Lindenmayer:** Conceptualization (equal); Funding acquisition (lead); Investigation (supporting); Writing‐review & editing (lead).

## Supporting information

Supplementary Material

## Data Availability

Data are provided in the supplementary material and from the Dryad Digital Repository (https://doi.org/10.5061/dryad.vmcvdncrh).
