# Supplementary material for: Can evolutionary theories of dispersal and senescence predict postrelease survival, dispersal, and body condition of a reintroduced threatened mammal?
Source: Ecol Evol. 2020 Dec 30;11(2):1002–12. doi: 10.1002/ece3.7115 (PMC7820150; doi:10.1002/ece3.7115)
Supplement: Supplementary file 1 — Supplementary Material [file ECE3-11-1002-s001.docx]

Supplementary Material

Table S1. Numbers of quolls, their sex and sanctuary of origin released at five different locations in 2018 and 2019

|  |  | Location | | | | | | | | | |  |
| --- | --- | --- | --- | --- | --- | --- | --- | --- | --- | --- | --- | --- |
| Year | Sanctuary | St Georges | | Botanic Gardens | | Steamers | | Furcraea | | Lone Pine | |  |
|  |  | F | M | F | M | F | M | F | M | F | M | Total |
| 2018 | Devils@Cradle | 6 | 4 |  |  |  |  |  |  |  |  | 10 |
|  | Trowunna | 4 | 6 |  |  |  |  |  |  |  |  | 10 |
| 2019 | Devils@Cradle |  |  | 8 | 5 |  |  |  |  |  |  | 13 |
|  | Trowunna |  |  |  |  | 5 | 5 |  |  |  |  | 10 |
|  | Aussie Ark |  |  |  |  |  |  | 3 | 5 | 3 | 6 | 17 |

Appendix S1: Terms used in statistical models

**Dispersal:** All models included log of the number of days tracked (based on earlier exploratory analysis) and combinations of the following terms: sex, sex-adjusted weight, year of release, sanctuary, and interactions between sex and year.

**Survival:** sex, sex-adjusted weight, year of release, sanctuary, generations-in-captivity and interactions between sex and year.

**Body condition:** sex, days since release (DSR), DSR^2, year of release, sanctuary and the following two-way interactions: sex x year, sex x DSR, sex x DSR^2, year x DSR, year x DSR^2 and three-way interactions: sex x year x DSR, sex x year x DSR^2.

Table S2: Widely applicable information criteria (WAIC) for each of the 17 models for the Generalized linear Gamma model for maximum dispersal. Where number of days tracked is denoted by NDT. The model with the lowest WAIC is given in bold.

| No | Model | WAIC | Delta  WAIC |
| --- | --- | --- | --- |
| 17 | log(NDT) + sex + weight + year+ sanctuary + sex x year | 145.87 | 5.03 |
| 16 | log(NDT) + sex + weight + year+ sanctuary | 142.91 | 2.07 |
| 15 | log(NDT) + sex + weight+ year | 149.01 | 8.17 |
| 14 | log(NDT) + sex + weight + sanctuary | 140.95 | 0.11 |
| 12 | log(NDT) + sex + year+ sanctuary | 147.82 | 6.98 |
| 11 | log(NDT) + weight + year+ sanctuary | 142.88 | 2.04 |
| 10 | log(NDT) + sex + weight | 149.77 | 8.93 |
| 9 | log(NDT) + sex + year | 149.33 | 8.49 |
| 8 | log(NDT) + weight + year | 147.15 | 6.31 |
| 7 | log(NDT) + sex + sanctuary | 146.23 | 5.39 |
| **6** | **log(NDT) + weight + sanctuary** | **140.84** | **0.00** |
| 5 | log(NDT) + year + sanctuary | 147.11 | 6.27 |
| 4 | log(NDT) + sex | 148.81 | 7.97 |
| 3 | log(NDT) + weight | 147.66 | 6.82 |
| 2 | log(NDT) + year | 147.09 | 6.25 |
| 1 | log(NDT) | 147.55 | 6.71 |

Table S3: Widely applicable information criteria (WAIC) for 32 possible models for the Cox proportional hazards model for survival. The model with the lowest WAIC is highlighted in bold.

| No | Model | WAIC | Delta WAIC |
| --- | --- | --- | --- |
| 33 | weight + sex + year + sanctuary + GensSinceWild + sex x year | 299.66 | 12.21 |
| 32 | weight + sex + year + sanctuary + GensSinceWild | 297.62 | 10.17 |
| 31 | weight + sex + year + sanctuary | 295.27 | 7.82 |
| 30 | weight + sex + year + GensSinceWild | 293.03 | 5.59 |
| 29 | weight + sex + sanctuary + GensSinceWild | 295.36 | 7.91 |
| 28 | weight + year + sanctuary + GensSinceWild | 297.79 | 10.34 |
| 27 | sex + year + sanctuary + GensSinceWild | 295.11 | 7.66 |
| 26 | year + sanctuary + GensSinceWild | 295.32 | 7.87 |
| 25 | sex + sanctuary + GensSinceWild | 293.07 | 5.62 |
| 24 | sex + year + GensSinceWild | 290.72 | 3.27 |
| 23 | sex + year + sanctuary | 293.07 | 5.63 |
| 22 | weight + sanctuary + GensSinceWild | 295.53 | 8.08 |
| 21 | weight + year + GensSinceWild | 293.87 | 6.42 |
| 20 | weight + year + sanctuary | 296.01 | 8.56 |
| 19 | weight + sex + GensSinceWild | 290.96 | 3.52 |
| 18 | weight + sex + sanctuary | 293.07 | 5.62 |
| 17 | weight + sex + year | 290.99 | 3.54 |
| 16 | sanctuary + GensSinceWild | 293.29 | 5.84 |
| 15 | year + GensSinceWild | 291.28 | 3.84 |
| 14 | year + sanctuary | 293.90 | 6.45 |
| 13 | sex + GensSinceWild | 288.68 | 1.23 |
| 12 | sex + sanctuary | 291.07 | 3.62 |
| 11 | sex + year | 289.43 | 1.98 |
| 10 | weight + GensSinceWild | 291.69 | 4.24 |
| 9 | weight sanctuary | 293.91 | 6.46 |
| 8 | weight + year | 291.99 | 4.54 |
| 7 | weight + sex | 289.00 | 1.55 |
| 6 | GensSinceWild | 289.23 | 1.78 |
| 5 | sanctuary | 291.88 | 4.43 |
| 4 | year | 290.02 | 2.58 |
| **3** | **sex** | **287.45** | **0.00** |
| 2 | weight | 289.96 | 2.51 |
| 1 | intercept | 288.04 | 0.59 |

Table S4a: Widely applicable information criteria (WAIC) for each of the interaction models for the multi-level model for weight. Where days since release is denoted by DSR. The model with the lowest WAIC is highlighted in bold.

| Model | WAIC |
| --- | --- |
| DSR + DSR^2 + year + sex + sanctuary | 1144.71 |
| DSR + DSR^2 + year + sex + sanctuary + sex x year + sex x DSR + sex x DSR^2 + year x DSR + year x DSR^2 | 1128.30 |
| **DSR + DSR^2 + year + sex + sanctuary + sex x year + sex x DSR + sex x DSR^2 + year x DSR + year x DSR^2 + sex x year x DSR + sex x year x DSR^2** | **1126.18** |
| DSR + DSR^2 + year + sex + sanctuary + sex x year + sex x DSR + sex x DSR^2 + year x DSR + year x DSR^2 + sex x year x DSR (*remove sex x year x DSR^2*) | 1492.76 |
| DSR + DSR^2 + year + sex + sex x year + sex x DSR + sex x DSR^2 + year x DSR + year x DSR^2 + sex x year x DSR + sex x year x DSR^2 (*remove Sanctuary*) | 1496.71 |
| DSR + DSR^2 + year + sex + sex x year + sex x DSR + sex x DSR^2 + year x DSR + year x DSR^2 + sex x year x DSR (*remove Sanctuary + sex x year x DSR^2*) | 1499.86 |

Table S4b: Model coefficients for best fitting multi-level model for weight. Where days since release is denoted by DSR. Sanctuary (Aussie Ark) is the baseline for sanctuary survival differences.

| Coefficient | Estimate | Lower  95% CI | Upper  95% CI | Lower  90% CI | Upper  90% CI |
| --- | --- | --- | --- | --- | --- |
| Intercept | 937.11 | 770.06 | 1104.12 | 799.67 | 1074.7 |
| DSR | -81.07 | -156.48 | -6.73 | -143.18 | -17.85 |
| DSR^2 | 74.76 | 12.61 | 135.71 | 23.76 | 124.9 |
| Year (2019) | -11.38 | -171.59 | 149.45 | -143.58 | 120.18 |
| Sex (Male) | 286.27 | 111.99 | 462.25 | 141.51 | 431.17 |
| Sanctuary (Devils Cradle) | -196.43 | -310.88 | -87 | -291.45 | -105.13 |
| Sanctuary (Trowunna) | -203.42 | -356.55 | -55.55 | -329.76 | -79.29 |
| Sex x DSR | -100.53 | -210.8 | 7.35 | -192.16 | -8.2 |
| Year x DSR | 66.27 | -18.51 | 154.7 | -4.62 | 139.41 |
| Sex x DSR^2 | 73.32 | -20.51 | 163.63 | -3.00 | 150.51 |
| Year x DSR^2 | -42.81 | -124.84 | 37.28 | -110.39 | 22.82 |
| Sex x Year | 98.39 | -111.12 | 314.14 | -80.54 | 276.7 |
| Sex x Year x DSR | 39.34 | -86.42 | 163.58 | -66.86 | 142.71 |
| Sex x Year x DSR^2 | -106.37 | -221.72 | 11.51 | -203.98 | -8.51 |
| Quoll Random Effect (SD) | 106 | 74.51 | 147.12 | 78.95 | 139.54 |
| Residual (SD) | 78.84 | 65.10 | 95.42 | 67.01 | 92.54 |
